# Polymorphisms in Vitamin D Receptor Genes in Association with Childhood Autism Spectrum Disorder

**DOI:** 10.1155/2018/7862892

**Published:** 2018-01-11

**Authors:** Zengyu Zhang, Sufang Li, Lianfang Yu, Jun Liu

**Affiliations:** ^1^Department of Pediatrics, Xiaoshan First People's Hospital, Hangzhou, Zhejiang 311201, China; ^2^Department of Clinical Laboratory, Zhejiang Xiaoshan Hospital, Hangzhou, Zhejiang 311202, China

## Abstract

Both genetic and environmental factors have been implicated in the etiology of autism spectrum disorder (ASD). This case-control study aimed to determine the association of single-nucleotide polymorphisms (SNPs) rs731276 (TaqI), rs1568820 (Cdx2), rs1544410 (BsmI), and rs2228570 (FokI) in the vitamin D receptor (VDR) gene with susceptibility of childhood ASD and severity of the disease. A total of 201 children with ASD and 200 healthy controls from the Han Chinese population were recruited. SNP genotyping was carried out by TaqMan probe-based real-time PCR using genomic DNA extracted from blood cells. Among four examined SNPs, only the CT genotype (odds ratio (OR) = 1.96, 95% confidence interval (CI) = 1.05–3.68, *P* = 0.0351) and the C allele (OR = 1.88, 95% CI = 1.02–3.46, *P* = 0.0416) of the rs731276 were significantly associated with increased risks of childhood ASD. None of the SNPs were associated with severity of childhood ASD. Our results reveal that certain polymorphisms in the VDR gene are a risk factor related to childhood ASD in the Han Chinese population.

## 1. Introduction

Autism spectrum disorder (ASD) is a neurodevelopmental disorder characterized by difficulty in social interactions, challenges in language and speech, and a tendency of repetitive behaviors [1]. Though the etiology accounting for ASD remains unknown, both genetic and environmental factors have been implicated as major risk factors of the disease [2–4]. Identification of these factors may assist with unraveling the etiology and develop novel prevention and treatment for the disease.

As a steroid hormone, vitamin D is known to be essential for calcium hemostasis and bone health. It also plays important roles in neural differentiation, immune modulation, antioxidation, and neurotrophic and neuroprotective actions, all of which are critical for embryogenesis and neurodevelopment [5–8]. The malfunctions of the vitamin D pathway may thus be attributable to the development of ASD. Previous studies have reported an association between vitamin D and ASD. Low serum vitamin 25(OH)D levels were associated with an increased risk of ASD [9, 10]. Vitamin D deficiency during pregnancy or early childhood was considered to be a risk factor for ASD [11, 12]. Supplementation of vitamin D for mothers of children with ASD during pregnancy and for their newborn siblings reduced the recurrence rate [13]. The varied prevalence of ASD in children born in different seasons, latitudes, and with varied skin pigmentation implied the role of vitamin D in the development of the disease [14]. The offspring exposed to vitamin D deficiency during gestation displayed autism-relevant structural and functional abnormalities in the brain and behavior, the same as those in animal studies [15, 16].

Vitamin D transmits signaling through binding to its receptor (VDR). The VDR gene is located on chromosome 12q13 consisting of nine exons and eight introns [17]. Several SNPs have been identified in this gene. The rs731236 (Taq1) is positioned at exon 9. This polymorphism changes protein structure and alters the binding specificity of vitamin D [18]. The VDR rs11568820 (Cdx2) is located in the promoter region. This polymorphism may influence the transcriptional activity [19]. The rs1544410 (BsmI) at intron 8 affects gene expression through regulation of mRNA stability [20]. The rs2228570 (FokI) is positioned at the start codon of exon 2. This polymorphism alters the initiation sites [18] and consequently generates two different-sized proteins [21]. Polymorphisms of VDR may also be involved in the development of ASD by influencing the functions of vitamin D pathway.

The role of VDR polymorphisms in the development of ASD has not been well studied. The results are still inconclusive [22, 23]. In this case-control study, the correlation between SNPs rs731276, rs1568820, rs1544410, and rs2228570 in the VDR gene and childhood ASD and its severity was examined in a Han Chinese population.

## 2. Materials and Methods

From September 2012 to June 2016, 201 Han Chinese children affected with ASD were recruited from hospitals in the Xiaoshan District of Zhejiang Province. Age- and gender-matched healthy Han Chinese children (*n* = 200) were recruited from both a preschool and a primary school in the same district [24]. This study was approved by the Medical Ethics Committee of Zhejiang Xiaoshan Hospital. Informed consent was obtained from parents or guardians of all children.

DNA was extracted from blood cells using the Qiagen Blood DNA Mini kit (QIAGEN China (Shanghai) Co. Ltd, Shanghai, China) following the protocol described previously [24, 25].

The data was analyzed using SAS 9.3 software (SAS Institute Inc., Cary, NC), and *P* values < 0.05 were considered statistically significant. The *χ*^2^ analysis was applied for the Hardy-Weinberg equilibrium test. Logistic regression analysis was applied to determine the relationship between SNPs and the risk of childhood ASD or severity of the disease. Odds ratios (ORs) and 95% confidence intervals (CIs) were calculated. Linkage disequilibriums (LD) among rs731276, rs1568820, rs1544410, and rs2228570 were analyzed using the SHEsis computer program [26].

## 3. Results

Genotype distributions of SNP rs731276, rs1568820, rs1544410, and rs2228570 were all in accordance with Hardy-Weinberg genetic equilibrium in both the control and case groups (Table 1).

Instead of three genotypes for each SNP, our results showed only two genotypes within this population, TT and CT of rs731276 and GG and AG of rs1544410. Logistic regression analysis showed that the CT genotype of rs731276 (OR = 1.96, 95% CI = 1.05–3.68, *P* = 0.0351) and the C allele of the rs731276 were significantly associated with an increased risk of childhood ASD (OR = 1.88, 95% CI = 1.02–3.46, *P* = 0.0416). There were no significant differences in the genotype distributions and allele frequencies between the case and control groups for the other three SNPs (Table 2).

The relationship between these SNPs and childhood ASD was further analyzed using dominant and recessive models for SNPs rs1568820 and rs2228570. No significant difference in genotypes of either SNP between children with ASD and healthy controls was observed in any of the models (Table 3).

These children affected with ASD were classified into mild-to-moderate and severe groups based on the scores of the Childhood Autism Rating Scale (CARS). There were 122 children with mild-to-moderate ASD and 79 with severe ASD. Logistic regression analysis showed that there was no significant association of these studied SNPs in the VDR gene regarding the severity of childhood ASD (Table 4).

**Figure 1:**
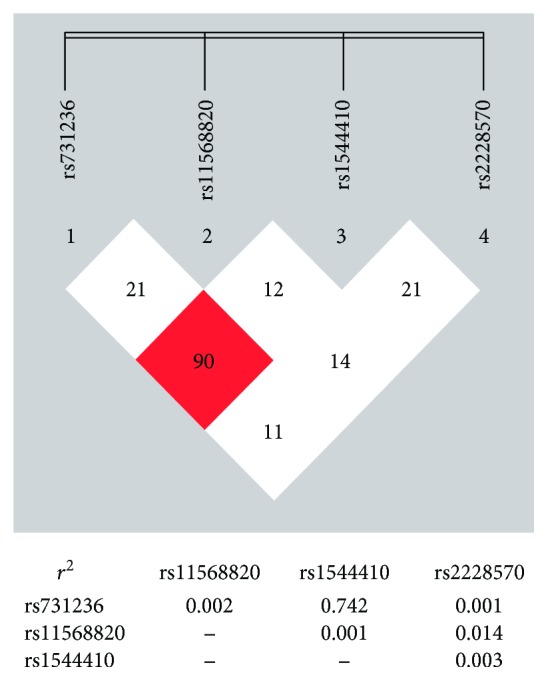
Linkage disequilibrium plot of the four examined SNPs in the VDR gene. The coefficient (*D*′) of the linkage disequilibrium was displayed as the number in the squares at the upper diagrams. The regression coefficient (*r*^2^) of the linkage disequilibrium was displayed at the bottom. The square in red indicates high linkage disequilibrium.

Pair-wise tests showed a strong linkage disequilibrium in between VDR rs731276 and rs1544410 (*D*′ = 0.90, *r*^2^ = 0.74), but not among other SNPs (Figure 1). There was no significant correlation between haplotypes (T-G and C-A) of rs731276-rs1544410 and risk of childhood ASD (Table 5).

## 4. Discussion

In this case-control study, we determined the relationship between SNPs in VDR genes and childhood ASD and its severity in a Han Chinese population. Our results showed that the CT genotype and C allele of VDR rs731276 were significantly associated with an increased risk of childhood ASD. There was no significant difference in genotype and allele frequencies of SNPs rs1568820, rs1544410, and rs2228570 between children with ASD and healthy controls. None of the studied SNPs were associated with severity of childhood ASD.

Two previous studies have thus far reported an association between certain SNPs in the VDR gene and ASD. Schmidt et al. examined SNPs in VDR genes of maternal, paternal, and child samples from children participating in the population-based CHARGE (CHildhood Autism Risks from Genetics and the Environment) case-control study. Their results showed that the paternal CC genotype of rs731236 and the AA genotype of rs1544410, individually or in combination, were significantly associated with an increased risk for ASD. However, no significant association between the above two SNPs and ASD was observed in autistic children or their mothers [22]. In a separate study, the SNPs in the VDR gene was examined among 237 children with ASD and 243 healthy controls from the Turkish population. Their data showed that the CC genotype of rs731236, the TT genotype of rs2228570, and the AA genotype of rs1544410 were significantly associated with increased risk of childhood ASD. The genotype of rs7975232 was not a significant risk factor for childhood ASD. The frequency of haplotype GTTT of rs1544410-rs731236-rs2228570-rs7975232 was significantly higher in children with ASD than in controls. The frequencies of the ATCG and GTCT haplotypes were statistically lower in children with ASD compared to healthy controls [23]. These two studies suggest that polymorphisms in the VDR gene may be associated with the risk of ASD, whereas different populations may also have their specific ASD-related SNPs.

Our results showed that both the CT genotype and the C allele of rs731236 in VDR were associated with increased risk of childhood ASD in this Han Chinese population. There were no significant differences in genotypes and allele frequencies of the SNPs rs1568820, rs1544410, and rs2228570 between children with ASD and healthy controls. No association between all four SNPs and the severity of childhood ASD was observed in this study. In addition, the rs731236 and rs1544410 showed a high linkage disequilibrium. However, both haplotypes T-C and C-T of rs731236-rs1544410 were not significantly associated with childhood ASD. Interestingly, our study showed that only two genotypes of Taq1 and Rs1544410 were observed in this Han Chinese population. This finding is consistent with the results of a previous study in a Chinese population [27]. Our results provide strong evidence that certain polymorphisms in the VDR gene are associated with risk of childhood ASD. It is essential to identify population-specific SNPs related to childhood ASD.

The polymorphisms in VDR genes have been correlated with serum 25(OH)D levels. Surprisingly, the genotypes or alleles related to a higher risk of ASD are associated with increased concentration of 25(OH)D. Coskun et al. discovered that the TT genotype of rs2228570 was significantly associated with increased risk of childhood ASD and with higher serum 25(OH)D in children with ASD [23]. Inconsistent with this finding, carriers of the TT genotype had significantly higher serum 25(OH)D concentrations in multiple sclerosis patients [28, 29]. Increased serum 25(OH)D levels in patients with the variant TT genotype may be a compensatory response due to reduced VDR activity [23]. In addition, both the Coskun group and our group identified that the C allele of rs731236 of the VDR gene was associated with increased risk of childhood ASD. In healthy subjects, from a Turkish cohort, the C allele carriers for rs731236 of the VDR gene had significantly higher levels of serum 25(OH)D [30]. In contrast, low levels of serum 25(OH)D were associated with an increased risk of ASD [9, 10]. It will be of clinical significance to study the role of the interaction between VDR polymorphisms and vitamin D status in the development of ASD.

Polymorphisms in the VDR gene have been associated with risks for many other neuropsychiatric disorders. SNPs rs731236 and rs1544410 in the VDR gene were associated with risk of multiple sclerosis in Mexican [31] and Kuwaiti studies [32]. A meta-analysis revealed that rs1544410 and rs2228570 were associated with susceptibility to Parkinson's disease and rs731236 was related to with Alzheimer's disease [33]. SNP rs2228570 was found to be significantly associated with cognitive decline in Parkinson's disease [34]. Jiang et al., reported that SNPs rs2228570 and rs7975232 were risk factors for childhood temporal lobe epilepsy [35]. No association of rs2228570 in the VDR gene with multiple sclerosis was found in other studies [29, 36]. SNPs rs7975232, rs731236, and rs1544410 in the VDR gene are not a predictor for disease disability progression rate in multiple sclerosis in Slovaks [37]. No correlation between Parkinson's disease and the VDR polymorphisms, including rs1544410, rs2228570, rs7975232, and rs731236, was found in a Korean population [38].

Limitations of this case-control study include a relatively small sample size. Only 4 SNPs of the VDR gene were determined, but no blood markers of vitamin D or calcium were examined in this study. A much higher proportion of boys with ASD was recruited in our study, with a ratio nearly 11 : 1 of boys to girls. Furthermore, all children with ASD enrolled in this study were patients being treated in the hospitals of the Xiaoshan District in Zhejiang Province. These patients may not be representative of all children with ASD from the general population of Chinese Han.

## 5. Conclusion

Our results suggest that certain polymorphisms in the VDR gene may cause susceptibility to the development of childhood ASD. More validation studies, with large sample sizes, are needed to validate the findings revealed in this study.

## Figures and Tables

**Table 1:** Hardy-Weinberg equilibrium tests (*P* values) for SNPs in the case and control groups.

SNPs	Cases	Controls
rs731276	0.2362	0.5302
rs1568820	0.1536	0.9113
rs1544410	0.3680	0.4542
rs2228570	0.9537	0.3961

**Table 2:** Distribution of SNP genotypes and allele frequencies among children with ASD and controls.

SNPs	Genotype/allele	Cases *n*^∗^ (%)	Controls *n* (%)	OR	95% CI	*P* value
rs731276						
	TT	170 (84.6)	183 (91.5)	1		
	CT	31 (15.4)	17 (8.5)	1.96	1.05–1.3.68	0.0351
	T	371 (92.3)	383 (95.7)	1		
	C	31 (7.7)	17 (4.3)	1.88	1.02–3.46	0.0416

rs1568820						
	GG	76 (38.2)	68 (34.2)	1		
	GA	86 (43.2)	96 (48.2)	0.80	0.52–1.24	0.3225
	AA	37 (18.6)	35 (17.6)	0.95	0.54–1.67	0.8472
	G	238 (59.8)	232 (58.3)	1		
	A	160 (40.2)	166 (41.7)	0.94	0.71–1.25	0.6654

rs1544410						
	GG	177 (88.1)	178 (89.9)	1		
	GA	24 (11.9)	20 (10.1)	1.20	0.64–2.26	0.5590
	G	378 (94.0)	376 (95.0)	1		
	A	24 (6.0)	20 (5.0)	1.19	0.65–2.20	0.5708

rs2228570						
	TT	53 (26.6)	40 (20.3)	1		
	CT	99 (49.8)	104 (26.9)	0.72	0.44–1.18	0.1897
	CC	47 (23.6)	53 (52.8)	0.67	0.38–1.18	0.1659
	T	205 (51.5)	184 (46.7)	1		
	C	193 (48.5)	210 (53.3)	0.83	0.62–1.09	0.1766

^∗^Number of cases and controls varied because some samples were unable to be genotyped.

**Table 3:** SNP genotype distributions and risk assessments for childhood ASD using genetic models.

SNPs/models	Genotype	Cases *n* (%)	Controls *n* (%)	OR	95% CI	*P* value
rs1568820						
Dominant	GG	76 (37.8)	68 (34.0)	1		
	AG + AA	125 (62.2)	132 (66.0)	0.84	0.56–1.28	0.4269
Recessive	GG + AG	164 (81.6)	165 (82.5)	1		
	AA	37 (18.4)	35 (17.5)	1.06	0.64–1.77	0.8128

rs2228570						
Dominant	TT	53 (26.6)	40 (20.3)	1		
	CC + CT	146 (73.4)	157 (79.7)	0.70	0.44–1.12	0.1384
Recessive	TT + CT	154 (76.6)	144 (73.1)	1		
	CC	47 (23.4)	53 (26.9)	0.83	0.53–1.31	0.4188

**Table 4:** Correlation between SNP genotypes and allele frequencies with severity of childhood ASD.

SNPs	Genotype/allele	Severe *n* (%)	Mild–moderate *n* (%)	OR	95% CI	*P* value
rs731276						
	TT	67 (84.8)	103 (84.4)	1		
	CT	12 (15.2)	19 (15.6)	0.97	0.44–2.13	0.9415
	T	146 (92.4)	225 (92.2)	1		
	C	12 (7.6)	19 (7.8)	0.97	0.46–2.07	0.9440

rs1568820						
	GG	29 (37.2)	47 (38.8)	1		
	GA	38 (48.7)	48 (39.7)	1.28	0.68–2.41	0.4372
	AA	11 (14.1)	26 (21.5)	0.69	0.30–1.59	0.3806
	G	96 (61.5)	142 (58.7)	1		
	A	60 (38.5)	100 (41.3)	0.89	0.59–1.34	0.5699

rs1544410						
	GG	70 (88.6)	107 (87.7)	1		
	GA	9 (11.4)	15 (12.3)	0.92	0.38–2.21	0.8472
	G	149 (94.3)	229 (93.9)	1		
	A	9 (5.7)	15 (6.1)	0.92	0.39–2.16	0.8532

rs2228570						
	TT	22 (28.2)	31 (25.6)	1		
	CT	38 (48.7)	61 (50.4)	0.88	0.39–1.95	0.7072
	CC	18 (23.1)	29 (24.0)	0.88	0.45–1.73	0.7436
	T	82 (52.6)	123 (50.8)	1		
	C	74 (47.4)	119 (49.2)	0.93	0.62–1.40	0.7350

**Table 5:** Haplotype distributions and corresponding risk assessments for childhood ASD.

Haplotype	Cases *n* (%)	Controls *n* (%)	OR	95% CI	*P* value
rs731276-rs1544410	*D*′ = 0.90, *r*^2^ = 0.74				
T-G	371 (92.3)	375 (94.7)	0.66	0.35–1.26	0.2051
C-A	24 (6.0)	16 (4.0)	1.52	0.79–2.90	0.2050

## Additional Points

*Summary Statement*. Certain polymorphisms in the vitamin D receptor gene are a risk factor for the childhood autism spectrum disorder. This finding facilitates our understanding of the genetic mechanisms of the disease.

## Abbreviations

ASD: Autism spectrum disorder
CARS: Childhood Autism Rating Scale
LD: Linkage disequilibriums
SNP: Single-nucleotide polymorphism
VDR: Vitamin D receptor.
